# Supplementary material for: New horizons in live and dehydrated black soldier fly larvae usage: Behavioral and welfare implications in “Bianca di Saluzzo” cockerels
Source: PLoS One. 2025 Feb 21;20(2):e0318793. doi: 10.1371/journal.pone.0318793 (PMC11844914; doi:10.1371/journal.pone.0318793)
Supplement: S1 File — (DOCX) [file pone.0318793.s001.docx]

**Video A**, wing flapping behavior of Bianca di Saluzzo males’ chicken available at:

<https://zenodo.org/records/12200725#:~:text=10.5281/zenodo.12200724>

**Video B**, raised huckle behavior of Bianca di Saluzzo males’ chicken available at:

<https://zenodo.org/records/12200803#:~:text=10.5281/zenodo.12200802>

**Video C**, aggressive pecking behavior of Bianca di Saluzzo males’ chicken available at:

<https://zenodo.org/records/12200840#:~:text=10.5281/zenodo.12200839>

**Video D**, sparring/fighting pecking behavior of Bianca di Saluzzo males’ chicken available at:

<https://zenodo.org/records/12200861#:~:text=10.5281/zenodo.12200860>

**Video E**, Plate/larvae exploration pecking behavior of Bianca di Saluzzo males’ chicken available at:

<https://zenodo.org/records/12200874#:~:text=10.5281/zenodo.12200873>
