# Supplementary material for: New horizons in live and dehydrated black soldier fly larvae usage: Behavioral and welfare implications in “Bianca di Saluzzo” cockerels
Source: PLoS One. 2025 Feb 21;20(2):e0318793. doi: 10.1371/journal.pone.0318793 (PMC11844914; doi:10.1371/journal.pone.0318793)
Supplement: Attachment — Submitted filename: Revewer comments.docx [file pone.0318793.s002.docx]

Reviewer 1	recommended: Minor Revision

The study examines the effects of live and dehydrated black soldier fly larvae on behavioral and
welfare implications in “Bianca di Saluzzo” cockerels. The paper is well-written and provides valuable insights into the efficiency of environmental enrichment with black soldier fly larvae in slow-growing male chickens. However, there are some points that need further attention.
- Why only male birds were used in this study?

L 25: “excreta corticosterone metabolites," which metabolites do the authors mean?

L 36-37: “However, the effects of live and dehydrated larvae (LL and DL, respectively)
supplementation on slow-growing male chicken welfare have never been formerly investigated.”. Add this sentence to the final paragraph of the introduction to highlight the research gap and to express the innovation of the work.

L 114-118: Have the authors analyzed the chemical composition of black soldier fly larvae? For the basal diet, what were the feed ingredients and chemical composition?

218: “2 birds/pen (48 birds in total)” With 6 replicates and 2 birds sampled from each replicated pen, there will be 36 birds in total not 48.

L 374-375: What were the reasons behind the better performance in BSFL-fed birds compared with control, despite the higher H/L ratio?

L 539-558: The conclusion should be more concise, emphasizing the practical outcomes of the study.

**Reviewer 2 recommended minor revision**

Strictly address the following points:

1. in the introduction section, need of the study should be ideally raised followed by the hypothesis of the study.

2. in materials and methods section, bird's husbandry should be more elaborated

3. in statistical analysis section, add mathematical model for better understanding of the data analysis.

4. in results section, add actual p-value of each result.

5. in discussion section, add logical reasoning of each result before discussing with the previous studies.

6. in conclusion section, add limitations and implication of the study.
